# Unravelling the Lipids Content and the Fatty Acid Profiles of Eight Recently Described *Halophytophthora* Species and *H. avicennae* from the South Coast of Portugal

**DOI:** 10.3390/md21040227

**Published:** 2023-03-31

**Authors:** Cristiana Maia, Thomas Jung, Aschwin Engelen, Marília Horta Jung, Luísa Custódio

**Affiliations:** 1 Centre of Marine Sciences (CCMAR), University of Algarve, 8005-139 Faro, Portugal; cris17couto@gmail.com (C.M.); aengelen@ualg.pt (A.E.); 2 Phytophthora Research Centre, Department of Forest Protection and Wildlife Management, Faculty of Forestry and Wood Technology, Mendel University in Brno, 613 00 Brno, Czech Republic; thomas.jung@mendelu.cz (T.J.); marilia.jung@mendelu.cz (M.H.J.); 3 Phytophthora Research and Consultancy, 83131 Nußdorf, Germany

**Keywords:** marine oomycetes, FAMEs, lipids, marine biotechnology

## Abstract

In this study, mycelia of eight recently described species of *Halophytophthora* and *H. avicennae* collected in Southern Portugal were analysed for lipids and fatty acids (FA) content to evaluate their possible use as alternative sources of FAs and understand how each species FAs profile relates to their phylogenetic position. All species had a low lipid percentage (0.06% in *H. avicennae* to 0.28% in *H. frigida*). Subclade 6b species contained more lipids. All species produced monounsaturated (MUFA), polyunsaturated (PUFA) and saturated (SFA) FAs, the latter being most abundant in all species. *H. avicennae* had the highest FA variety and was the only producer of γ-linolenic acid, while *H. brevisporangia* produced the lowest number of FAs. The best producer of arachidonic acid (ARA) and eicosapentaenoic acid (EPA) was *H. thermoambigua* with 3.89% and 9.09% of total FAs, respectively. In all species, palmitic acid (SFA) was most abundant and among the MUFAs produced oleic acid had the highest relative percentage. Principal component analysis (PCA) showed partial segregation of species by phylogenetic clade and subclade based on their FA profile. *H. avicennae* (Clade 4) differed from all other Clade 6 species due to the production of γ-linolenic and lauric acids. Our results disclosed interesting FA profiles in the tested species, adequate for energy (biodiesel), pharmaceutical and food industries (bioactive FAs). Despite the low amounts of lipids produced, this can be boosted by manipulating culture growth conditions. The observed interspecific variations in FA production provide preliminary insights into an evolutionary background of its production.

## 1. Introduction

*Halophytophthora* species are oomycetes (kingdom Straminipila, family Peronosporaceae) commonly known to inhabit marine and estuarine ecosystems especially in tropical and subtropical areas [1,2,3]. However, *H. vesicula* (under its previous designation as *Phytophthora vesicula*) was first described from a marine habitat near Vancouver [4], and more recent surveys demonstrated the presence of *Halophytophthora* species in the North Sea [5,6]. Furthermore, Yang and Hong [7] reported a new species, *H. fluviatilis*, from a freshwater location in Virginia, USA, which was also found widespread in rivers and streams of Eastern Spain [8]. These findings indicate that this genus is well adapted to a wide range of temperature and salinity conditions. Halophytophthoras are mostly known as saprophytes, playing an important role in decomposition and secondary production [9]. However, some species have been isolated from seeds of the seagrass *Zostera marina* and are associated with reduced germination rates [6,10], suggesting they might also act as pathogens.

The genus *Halophytophthora* was first created to accommodate nine marine *Phytophthora* species that differed from those of freshwater and terrestrial origin in terms of morphological and cultivation characteristics, such as differences in the apical structure of the sporangium and the mode of zoospore release [11]. Since phylogenetic studies demonstrated that the genus was polyphyletic, numerous species were transferred to other genera, i.e., *Calycofera*, *Phytopythium*, *Salisapilia*, *Salispina*, leaving *H. vesicula* (the type species), *H. avicennae*, *H. batemanensis*, *H. fluviatilis*, *H. insularis*, *H. polymorphica*, *H. souzae* and eight newly described species from Portugal (*H. brevisporangia*, *H. celeris*, *H. frigida*, *H. lateralis*, *H. lusitanica*, *H. macrosporangia*, *H. sinuata*, *H. thermoambigua*) in the genus *Halophytophthora* sensu stricto [12,13,14,15,16,17,18].

Fatty acids (FAs) can be divided into saturated (SFAs), monounsaturated (MUFAs) and polyunsaturated (PUFAs) based on the absence or presence of one or more double bonds. FAs have, in general, multiple applications, ranging from human health improvement, and aquaculture, to biotechnological applications. For example, microorganisms capable of producing relevant amounts of SFAs are attractive for the biodiesel production industry and could be used to mitigate sustainability issues related to first- and second-generation biofuels [19,20]. PUFAs are divided into two major families, omega 3 (ω-3) and omega 6 (ω-6) [21,22] and have beneficial health effects in humans. For instance, arachidonic acid (ARA), an ω-6 FA, as well as docosahexaenoic acid (ω-3, DHA) are essential in the development of the central nervous system and the retina in infants, and ω-3 FA, in general, have anti-inflammatory and anti-angiogenic properties that can be used in anticancer therapies [23,24,25]. Traditionally, the main source of PUFAs is wild fish, but with the decrease in fish stocks due to overfishing, alternative sources are needed [21]. Among the sustainable alternatives are aquaculture fish, bioengineered plants, microalgae and krill [22]. Several aquatic microorganisms have also been explored, with microalgae and thraustochytrids giving promising results in terms of the production of PUFAs, in particular omega-3 FAs [26,27,28]. Oomycetes have mostly been overlooked, although several studies showed that members from several genera, including *Halophytophthora*, *Phytophthora*, *Pythium* and *Salispina*, can produce a wide variety of FAs [29,30,31,32,33]. Pang et al. [31] reported, for the first time, the production of arachidonic acid (ω-6, ARA) and eicosapentaenoic acid (ω-3, EPA) by different *Halophytophthora* species isolated from fallen mangrove leaves in Taiwan, suggesting these oomycetes as a potential alternative source of such compounds.

A survey conducted by our research group in 2015 along the Algarve coast resulted in the isolation of *H. avicennae* and eight previously unknown *Halophytophthora* taxa, which were recently described as new species [18]. As a result, the opportunity to expand the limited knowledge regarding FA production by Halophytophthoras emerged. All studies previously published on *Halophytophthora* FAs were conducted on isolates from mangroves in Taiwan or the Philippines [31,32,33,34,35], while this study aimed to bring new insights on FAs produced by all nine *Halophytopthora* species isolated along the Algarve coast from different ecosystems (salt marshes and estuaries), and to unravel interspecific differences in FA profiles and analyse how these relate to their phylogenetic position. Since there is variability in the production of FAs among different species and even isolates from the same species, it is of value to investigate the FA profiles of newly described species to screen for species that produce more interesting profiles. Furthermore, to evaluate the potential of Halophytophthoras to be used as an alternative source of interesting FAs, lipid production was analysed. To achieve this goal, we screened the nine *Halophytophthora* species for the production of SFAs, MUFAs and PUFAs, using a direct transesterification method for extraction and derivatisation, followed by gas chromatography coupled to mass spectrometry (GC/MS) for detection, and lipid accumulation using a modified gravimetric method. To analyse variations in FA profiles among the tested species, principal component analysis (PCA) was performed.

## 2. Results and Discussion

### 2.1. Total Lipids

**Figure 1:**
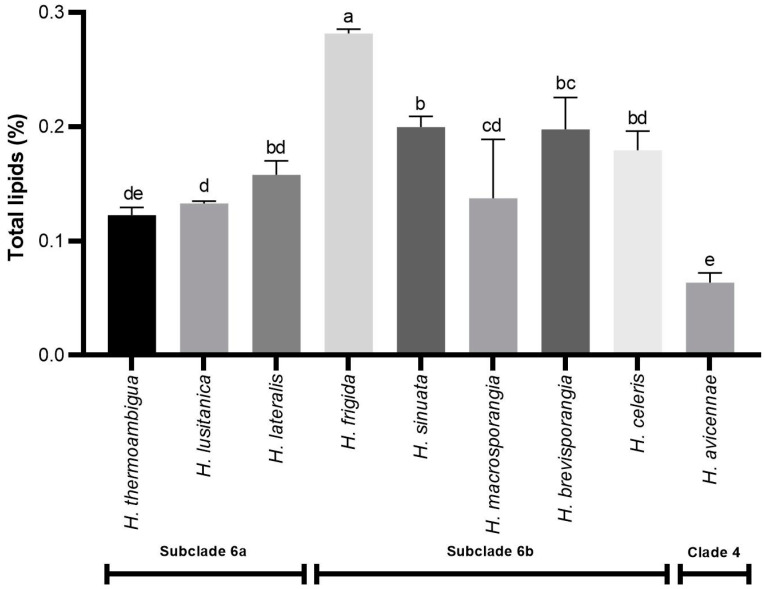
Percentage of total lipids in the nine *Halophytophthora* species tested. Results are means ± SD (*n* = 3) Columns marked with the same letter are not statistically different from each other (*p* ≥ 0.05).

All *Halophytophthora* species had low amounts of lipids with some visible interspecific variation. The percentage of total lipids ranged from 0.06% in *H. avicenniae* to 0.28% in *H. frigida*, both differing significantly from the other species (except for *H. avicennae* and *H. thermoambigua*) and from each other (Figure 1).

A recent phylogenetic analysis of all *Halophytophthora* species resulted in an updated phylogeny of the genus organized in 10 clades. All eight new *Halophytophthora* species from the Algarve coast reside in Clade 6 and are divided into Subclade 6a and Subclade 6b. *Halophytophthora avicennae* resides in Clade 4 [18]. There seems to be a tendency for Halophytophthoras from Subclade 6b, which includes all three homothallic species (*H. frigida*, *H. sinuata* and *H. macrosporangia*) and the two fastest growing species (*H. brevisporangia* and *H. celeris*), to have higher amounts of lipids, since four of the five species with the highest percentages of lipids are from this subclade (Figure 1). *Halophytophthora macrosporangia* is more similar to the species from Subclade 6a, but there was substantial variation in the lipid percentage among the three replicates of this species, ranging from 0.08% to 0.18% (av. 0.14 ± 0.05%). The lowest value, although not a significant outlier, differs considerably from the other two values and is closer to *H. avicennae*.

Homothallic species produce reproductive sexual structures (oogonia and antheridia) in single culture, i.e., without the need of a mating partner. Some studies suggest that the sexual structures have higher amounts of lipids than the vegetative ones. To our knowledge, there are no reports on total lipids in *Halophytophthora* spp., and only a few studies from more than 45 years ago were performed on *Phytophthora* species. Specifically, Bartnicki-Garcia [36] conducted a study on the chemistry of the hyphal walls of *P. cinnamomi* and *P. parasitica* revealing that both species had small amounts of lipids, mostly of the bound type (2.1% and 0.9%, respectively), while free lipids accounted only for 0.3% and 0.2%, respectively, of the wall dry weight. In 1973, Lippman et al. [37] studied the composition of the oospore and oogonium walls of *P. megasperma*. Lipids were extracted from the oospore–oogonium walls (OOW) and from the oospore–oogonium–antheridium (OOA) apparatus. The OOA presented the higher percentage of lipids (19.2%), mostly of the bound type. In both studies, lipids were extracted using a direct extraction method that was followed by acidic digestion for the extraction of the bound lipids. The mycelia of the three homothallic species used in this study, *H. frigida*, *H. macrosporangia* and *H. sinuata*, harvested after 10 days of growth in liquid broth, were not checked for the presence of oogonia and antheridia and, hence, it is not known whether or not these structures were produced. Furthermore, different species have different timings to produce sexual structures. In the case of *Phytophthora* species, which are closely related to *Halophytophthora*, the oospore, containing inside a large lipid-like body (the ooplast), is usually formed after 5 to 10 days of incubation. However, some species require longer periods of incubation [38]. It is possible that *H. frigida* produced the reproductive structures faster or more abundantly than the other two homothallic species, which could explain the higher level of lipids detected. *Halophytophthora sinuata*, the species with the second highest percentage of lipids, produces the largest oogonia of all known homothallic *Halophytophthora* species [18], which might explain the high lipid content. However, the lipid content of the sterile species *H. brevisporangia*, *H. lateralis* and *H. celeris* did not differ significantly from *H. sinuata* and were even higher than in the homothallic *H. macrosporangia* (Figure 1). Future studies are required to assess the production of oogonia and antheridia by homothallic *Halophytophthora* in liquid broth and the possible association between their abundance and the lipid contents of mycelia.

Apart from the presence of reproductive structures that might influence the concentration of lipids in *Halophytophthora*, there was no indication of any morphological and physiological characteristic explaining the higher percentage of lipids in isolates from Subclade 6b. For instance, *H. frigida* from Subclade 6b and *H. lateralis* and *H. lusitanica* from Subclade 6a have similar growth rates at 20 °C [18] but show significant differences in their lipid contents (Figure 1). Furthermore, even within Subclade 6b the lipid contents of *H. frigida* isolate BD675 and *H. macrosporangia* isolate BD656 differ significantly despite sharing a low optimum temperature for growth at 15 °C and generally slow growth (Figure 1) [18].

Our study shows that Halophytophthoras have low amounts of lipids. However, the lipid content can be increased through manipulation of the culture growing conditions, for example, by growing homothallic species for a longer period, allowing them to produce more sexual structures or by using alkaline or acid hydrolysis to extract the bound lipids that might not have been extracted. There are no studies conducted in *Halophytophthora* spp. for lipid accumulation improvement through culture conditions manipulation; however, it is known that in microalgae cultivation, inducing stress is a traditional way of increasing lipids via nutrient deficiency, salinity stress and temperature [39,40]. Assays are now being conducted in this regard.

### 2.2. FA Profile

All the *Halophytophthora* species produced SFAs, MUFAs and PUFAs (both ω-3 and ω-6) with, on average, ten FAs per species, but interspecific variation was observed in the number and amount of FAs detected (Table 1). SFAs were the most abundant group of FAs produced by all species, ranging between 43.68% in *H. sinuata* and 61.76% in *H. brevisporangia*, followed by PUFAs and, finally, MUFAs (Table 1).

**Figure 2:**
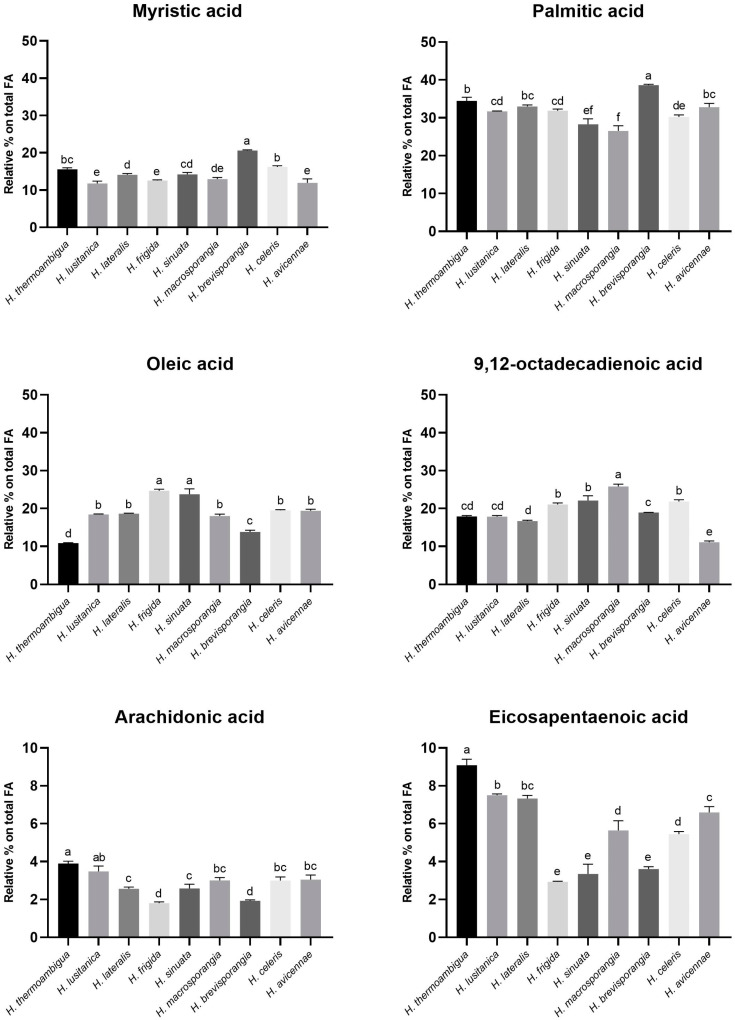
Relative percentages in total fatty acids of the two most abundant SFAs (Myristic and Palmitic acids), the three most abundant PUFAs (9,12-octadecadienoic, Arachidonic and Eicosapentaenoic acids) and the most abundant MUFA (Oleic acid). Results are means ± SD (*n* = 3). Columns marked with the same letter are not statistically different from each other (*p* ≥ 0.05).

*Halophytophthora avicennae* produced a wider variety of FAs (12), being the only species producing γ-linoleic acid (2.12% of total FAs), while *H. brevisporangia* produced the lowest number of FAs (7). The four most abundant FAs in all species were C16:0 (palmitic acid), C18:1n9c (oleic acid), C18:2n6 (9,12-octadecadienoic acid) and C14:0 (myristic acid) (Figure 2). Among the five PUFAs detected, 9,12-octadecadienoic acid was the most abundant, ranging between 11.11% in *H. avicennae* and 25.8% in *H. macrosporangia* (Figure 2). Interestingly, both eicosapentaenoic acid (EPA) and arachidonic acid (ARA) were produced by all species. EPA was produced in higher amounts than ARA, with *H. thermoambigua* being the most prolific producer with a percentage of 9.09%. The latter species also had the highest percentage of ARA (3.89%) which was statistically different from all other species, except for *H. lusitanica* (3.48%) (Figure 2). In the study of Pang et al. [31], the best ARA producer was *Salispina spinosa* (previously *H. spinosa* var. *spinosa*; isolate IBM 162) with 25.02%, while EPA was prevalent in *H. avicenniae* (isolate IBM 144) with 18.42%, approximately the double of our best producer, *H. thermoambigua*, and almost three times higher than the percentage observed for *H. avicennae* isolate BD697. Both PUFAs have high importance for human health. ARA is essential in the development of the central nervous system and the retina in infants [23], whereas EPA plays a major role in preventing cardiovascular diseases [41].

Besides PUFAs, SFAs and MUFAs were also produced. Although being generally less interesting in terms of human health improvement than PUFAs, with some of them even being associated with cardiovascular diseases, SFAs and MUFAs can have interesting biotechnological uses, for instance, in the production of biodiesel. The level of unsaturation correlates with the suitability of the FAs to be used as biodiesel. High levels of SFAs and MUFAs are desirable due to their oxidative and thermal stability [42]. As previously stated, SFAs were the most abundant group of FAs produced by all *Halophytophthora* species. Former studies with other marine oomycetes showed promising results regarding the potential use of these microorganisms as alternative sources of lipids for biodiesel production. For example, Patel et al. [43] studied the production of microbial oils in the aquatic oomycete *Achlya diffusa*. Cultivated in sugarcane bagasse, this water mold showed a total lipid content of 50.26% (*w*/*w*) and a FA profile with high levels of SFAs and MUFAs comparable to those of vegetable oils. Furthermore, some of the biodiesel properties of the lipids obtained from *A. diffusa*, such as density and kinematic viscosity, satisfied the limits as determined by international standards ASTM-D6751 and EN-14214, demonstrating its suitability as a fuel for diesel engines. Regarding SFAs, palmitic acid was most abundant, with relative percentages ranging between 26.56% (*H. macrosporangia*) and 38.63% (*H. brevisporangia*) (Figure 2). These results are in accordance with other studies on *Halophytophthora* species, where palmitic acid was the most abundant SFA and sometimes even being the most abundant of all FAs [31,34]. As with MUFAs, oleic acid had the highest relative percentage with *H. frigida* and *H. sinuata* being the best producers and differing significantly from the other species (Figure 2).

Our isolates did not produce docosahexaenoic acid (DHA), an important ω-3 FA which is essential for the development of brain function in infants and the maintenance of brain function in adults [25]. In contrast to all *Halophytophthora* isolates tested in the present study, isolate *H.* sp. S13005YL-3.1, belonging to an unknown *Halophytophthora* species, produced DHA when grown in PYG media but failed to produce EPA in any of the conditions tested in the study of Say et al. [32]. Culture conditions greatly affect mycelial growth and the production of FAs, and can be manipulated to optimize the production of these compounds. Duan et al. [30] studied the effect of different parameters, including growth medium, incubation temperature and time, on the production of FAs by *Phytophthora* and observed that these parameters have a significant impact on the production of those compounds. Caguimbal et al. [33] studied the FA profile of *Halophytophthora vesicula* (isolates AK1YB2 and PQ1YB3) and *Salispina spinosa* (isolate ST1YB3) using both V8S and PYGS media and their results showed significant differences in the production of individual FAs. PYGS proved to be a better medium to produce ARA in all three isolates tested. Say et al. [32] tested the production of FAs in media with different pH and salinity and concluded that isolate *H.* sp. S13005YL-3.1 produced a higher percentage of ARA when grown in V8 (pH 6; 10 ppt), compared to V8 (pH 8; 30 ppt). V8 media, like the one used in this study, PYG media and even a mix of both are the most widely used culture media in studies on the production of FAs by oomycetes and Thraustochytrids [31,32,33,34,35,44]. Owing to their production from commercially available vegetable juice, V8 media cannot as easily be manipulated as PYG, in which the concentrations of each component can be optimised to enhance FA production. Therefore, it seems likely that the FA production of the *Halophytophthora* species tested in our study could be increased by using different culture media and optimising important parameters such as salinity and pH.

Another potential use of FA produced by *Halophytophthora* species could be in cancer research. As mentioned before, the most abundant FAs in all *Halophytophthora* species tested in this study were palmitic, oleic, myristic and 9,12-octadecadienoic acids. These FA profiles resemble those from the species used in the study by Devanadera et al. [34], which explored the cytotoxic potential against breast adenocarcinoma cells (MCF7) of crude FAs produced by *Halophytophthora* and *Salispina* isolates. The crude FAs displayed significant toxicity towards MCF7 cells while being nontoxic to HDFn cells (normal dermal fibroblasts) as determined by the MTT assay, indicating that FAs, and particularly those produced by *Halophytophthora*, may potentially be used as adjuvants in cancer treatments.

**Figure 3:**
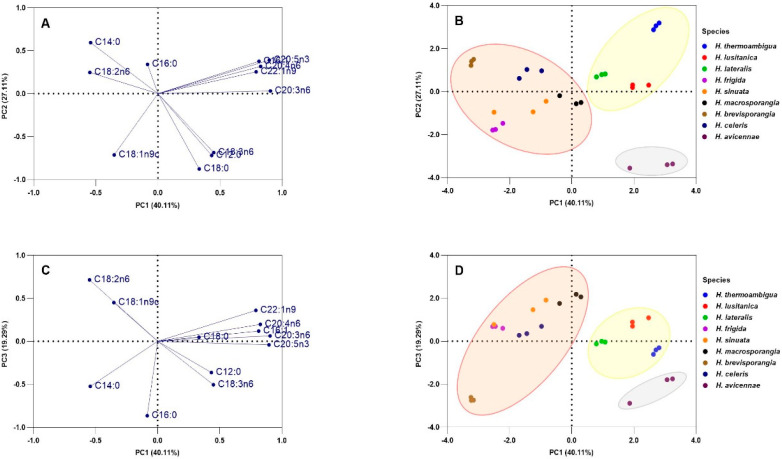
Principal component analysis (PCA) loading plots (**A**,**C**) and score plots (**B**,**D**) of PC1-PC2 (**A**,**B**) and PC1-PC3 (**C**,**D**). Proportions of variance are shown in brackets. Ovals in the score plots represent the phylogenetic clade and subclades of the species (Clade 4 in grey, Subclade 6a in yellow and Subclade 6b in coral). Dots with the same colour are replicates (*n* = 3).

PCA was used to identify variations in the FA profiles of all nine *Halophytophthora* species tested as well as potential relationships to their phylogenetic positions. Overall, PCA shows that *Halophytophthora* species can be separated, to some extent, based on their FA profiles. Three significant PCs were generated (eigenvalues higher than 1): PC1, PC2 and PC3 accounted for 40.11%, 27.11% and 19.29% of the total variance, respectively. According to the loading plots (Figure 3A,C), PC1 has strong positive contributions from primarily C:20 FA (arachidonic, eicosapentaenoic and eicosatrienoic acids) and negative contributions from 9,12-octadecadienoic acid (C18:2n6) and myristic acid (C14:0). PC2 is mostly negatively correlated with C18 FA (stearic, oleic and γ-linolenic acids) and lauric acid and positively correlated with myristic acid, while PC3 is positively correlated with oleic and 9,12-octadecadienoic acids and negatively correlated with myristic, palmitic and γ-linolenic acids. The score plots (Figure 3B,D) show *H. avicennae* from Clade 4 and the three *Halophytophthora* species from Subclade 6a on the positive PC1 axis, while species from Subclade 6b are mostly scattered on the negative PC1 axis, except for *H. macrosporangia*, where two of the replicates have positive values on the PC1 axis and the other replicate resides on the negative side.

The species *H. sinuata* and *H. frigida* are separated mainly because of the influence of oleic acid, of which they have the highest percentages compared to the other species. *Halophytophthora avicennae* from Clade 4 is clearly separated from the other species, mainly due to the production of γ-linolenic acid and lauric acid. *Halophytophthora brevisporangia* clusters separately from the other species mainly influenced by the production of myristic acid. In the PC1–PC2 loading plot (Figure 3C), which explains most of the variability, *H. thermoambigua* is differentiated from the other two Subclade 6a species mostly because of the low amounts of oleic acid it produces compared with the other two species. 

## 3. Materials and Methods

### 3.1. Halophytophthora Isolates

Using an in situ baiting technique, the nine *Halophytophthora* species and isolates used in this study were obtained in December 2015 from various marine and brackish-water ecosystems at six sites along the Algarve coast of Portugal [18]: *H. avicennae* isolate BD697 from the estuary of Rio Guadiana; *H. brevisporangia* isolate BD658 from a tidal pond in a salt marsh of the Ria Formosa at Quelfes; *H. celeris* isolate BD646 and *H. macrosporangia* isolate BD645 from a tidal channel in a salt marsh of the Ria Formosa at Santa Luzia; *H. frigida* isolate BD675 from a coastal lagoon of the Ria de Alvor; *H. lateralis* isolate BD680 from a coastal lagoon of the Ria Formosa at Almancil; *H. lusitanica* isolate BD632 and *H. thermoambigua* isolate BD631 from the estuary of Rio Séqua at Tavira; and *H. sinuata* isolate BD656 (=CBS 147237) from a tidal pond in a salt marsh of the Ria Formosa at Santa Luzia. Stock cultures were maintained on carrot juice agar (CA) at 10 °C in the dark [45].

### 3.2. Biomass Production

All *Halophytophthora* isolates were grown in 90 mm Petri dishes containing V8 juice agar (V8A) [46] for a week at 20 °C in the dark. V8A discs cut from the edge of the growing colonies were transferred to 90 mm Petri dishes with V8 juice broth (V8B; 3 g CaCO_3_, 100 mL V8 juice, 900 mL distilled water) for 10 days at 20 °C in the dark. Mycelia were harvested, washed thoroughly with sterilized distilled water, blotted dry with filter paper, and stored at −80 °C prior to lyophilisation. After lyophilisation, mycelia were ground to a powder using a mortar and pestle and stored in sealed tubes in the dark at 20 °C until total lipids and fatty acid analysis.

### 3.3. Total Lipids Quantification

Total lipids quantification was accomplished by using the modified gravimetric method of Bligh and Dyer [47]. Eight milligrams lyophilized *Halophytophthora* biomass was suspended in 0.8 mL distilled water and left to rest for 30 min. Then, 2 mL methanol and 1 mL chloroform were added, and the mixture homogenized in the Ultra-Turrax^®^ for 1 min, followed by adding 1 mL chloroform and homogenising for 30 s and finally by adding 1 mL distilled water and homogenising for 30 s. The tubes containing this mixture were centrifuged for 10 min at room temperature at. The bottom layer was collected, and a known amount (between 0.8 and 1.2 mL) was pipetted to previously weighed tubes that were then placed in a dry bath inside the fume hood overnight. The tubes were weighed after evaporation of the chloroform. Total lipids were quantified as a percentage of dry weight.

### 3.4. Fatty Acid (FA) Recovery and Analysis

FA recovery was achieved using the modified direct transesterification method of Lepage and Roy [48]. One hundred milligrams *Halophytophthora* biomass was suspended in the solvent solution (methanol/acetyl chloride, 20:1, *v*/*v*), and cell disruption was achieved using an ultrasonic bath for 15 min followed by the addition of 1 mL hexane and 1 h heating at 100 °C. Afterward, the mixture was chilled in an ice bath and 1 mL distilled water was added to stop the reaction. Finally, 3 mL hexane was added, vortexed and the organic top layer, containing the fatty acid methyl esters (FAMEs), was removed. After repeating the last steps twice, the fractions were merged and then evaporated overnight on a hot plate at 60 °C. After evaporation, FAMEs were reconstituted in hexane at a concentration of 2 mg mL^−1^. FAMEs were analysed in a Bruker Scion 456/GC, Scion TQ MS, coupled with a 30 mm ZB-5MS capillary column (0.25 mm internal diameter and 0.25 µm film thickness, Phenomenex, Torrance, CA, USA) using helium as carrier gas (1 mL min^−1^). Samples (1 µL) were injected at 300 °C in splitless mode and the following temperature profile of the GC oven: 60 °C (1 min), 30 °C min^−1^ to 120 °C, 4 °C min^−1^ to 250 °C, and 20 °C min^−1^ to 300 °C (4 min). The identification of the FAMEs was performed by using a Supelco^®^ 37 Component FAME Mix (Sigma-Aldrich, Sintra, Portugal). Results are expressed as percentages of total FA.

### 3.5. Statistical Analysis

Results were expressed as means ± standard deviation (SD) and the experiments were performed in triplicate. One-way ANOVA was used to analyse total lipids and fatty acids data, followed by Tukey’s test for assessing significance of differences. Assumptions of normality and homogeneity of variance were tested using the Shapiro–Wilk and the Brown–Forsythe tests, respectively. Principal component analysis (PCA) was performed to assess FA contribution to interspecies variation. The statistical software used to perform the analysis was GraphPad Prism version 9 for Windows (GraphPad Software, San Diego, CA, USA).

## 4. Conclusions

This is the first study unravelling the FA production by eight recently described *Halophytophthora* species and *H. avicennae* from marine and brackish-water ecosystems in Southern Portugal. It was shown that all isolates accumulate lipids and produce a wide range of interesting FAs, similar to those produced by other oomycetes previously examined for their potential use as alternative FA sources. All the *Halophytophthora* species produced SFAs (the most abundant), followed by PUFAs and, finally, MUFAs, with on average ten FAs per species. To improve the process and yield, further research is needed. For example, cultivation of the isolates in different media at different pH and salinity conditions, and a different extraction technique should be tested to increase the amount of lipids produced and accumulated by *Halophytophthora* spp. Multivariate analysis revealed variations in FA production among *Halophytophthora* species, providing preliminary insights into an evolutionary background of FA production.

## Figures and Tables

**Table 1:** Relative composition of fatty acids (FAs) in nine *Halophytophthora* species (% of total amount of FAs).

Fatty Acids ^1^	*Halophytophthora* Species and Isolate Codes								
	*H. thermoam-bigua* BD631	*H. lusitanica* BD632	*H. lateralis* BD680	*H. frigida* BD675	*H. sinuata* BD656	*H. macrospo-rangia* BD645	*H. brevispo-rangia* BD658	*H. celeris* BD646	*H. avicennae* BD697
**C12:0** **Lauric acid**	nd	nd	nd	nd	0.34 ± 0.59	0.89 ± 0.78	nd	nd	3.95 ± 0.23
**C14:0** **Myristic acid**	15.56 ± 0.42	11.76 ± 0.64	14.09 ± 0.30	12.55 ± 017	14.18 ± 0.52	12.93 ± 0.44	20.61 ± 0.15	16.17 ± 0.33	11.93 ± 1.04
**C16:0** **Palmitic acid**	34.50 ± 0.95	31.66 ± 0.16	33.01 ± 0.40	31.86 ± 0.45	28.32 ± 1.40	26.56 ± 1.35	38.63 ± 0.22	30.23 ± 0.55	32.82 ± 0.99
**C18:0** **Stearic acid**	2.52 ± 0.15	4.07 ± 0.04	3.11 ± 0.02	4.28 ± 0.10	3.34 ± 0.29	4.11 ± 0.03	2.52 ± 0.09	2.40 ± 0.19	5.37 ± 0.15
**∑ SFA**	52.58	47.50	50.21	48.70	43.68	44.99	61.76	48.80	54.06
**C16:1** **Palmitoleic acid**	2.39 ± 0.01	1.24 ± 0.08	1.39 ± 0.02	0.61 ± 0.04	0.66 ± 0.08	0.86 ± 0.06	nd	0.98 ± 0.07	1.14 ± 0.04
**C18:1n9c** **Oleic acid**	10.86 ± 0.11	18.45 ± 0.09	18.66 ± 0.10	24.69 ± 0.42	23.76 ± 1.42	18.03 ± 0.48	13.79 ± 0.49	19.53 ± 0.14	19.42 ± 0.38
**C22:1n9** **Erucic acid**	1.72 ± 0.03	1.61 ± 0.18	1.32 ± 0.10	0.22 ± 0.13	0.83 ± 0.03	1.19 ± 0.05	nd	0.40 ± 0.70	0.87 ± 0.11
**∑ MUFA**	14.97	21.31	21.37	25.52	25.25	20.07	13.79	20.92	21.42
**C18:2n6** **9,12-octadecadienoic acid**	17.89 ± 0.25	17.85 ± 0.29	16.71 ± 0.20	21.05 ± 0.43	22.11 ± 1.25	25.80 ± 0.64	18.92 ± 0.07	21.83 ± 0.48	11.11 ± 0.35
**C18:3n6** **γ-linolenic acid**	nd	nd	nd	nd	nd	nd	nd	nd	2.12 ± 0.11
**C20:3n6** **Eicosatrienoic acid**	1.58 ± 0.05	2.36 ± 0.20	1.82 ± 0.17	nd	0.52 ± 0.45	0.98 ± 0.05	nd	nd	1.65 ± 0.29
**C20:4n6** **Arachidonic acid (ARA)**	3.89 ± 0.12	3.48 ± 0.29	2.57 ± 0.09	1.80 ± 0.07	2.58 ± 0.22	3.00 ± 0.15	1.93 ± 0.06	3.00 ± 0.19	3.05 ± 0.24
**C20:5n3** **Eicosapentaenoic acid (EPA)**	9.09 ± 0.31	7.50 ± 0.06	7.33 ± 0.17	2.93 ± 0.03	3.35 ± 0.52	5.64 ± 0.52	3.60 ± 0.13	5.44 ± 0.15	6.59 ± 0.31
**∑ PUFA**	32.45	31.20	28.42	25.78	28.56	35.43	24.45	30.28	24.52

^1^ SFA = saturated FA, MUFA = monounsaturated FA, PUFA = polyunsaturated FA. Values represent means ± SD (*n* = 3); nd = not detected.
